# Levomepromazine and clozapine induce the main human cytochrome P450 drug metabolizing enzyme CYP3A4

**DOI:** 10.1007/s43440-020-00157-4

**Published:** 2020-09-04

**Authors:** Przemysław J. Danek, Agnieszka Basińska-Ziobroń, Jacek Wójcikowski, Władysława A. Daniel

**Affiliations:** Department of Pharmacokinetics and Drug Metabolism, Maj Institute of Pharmacology, Polish Academy of Sciences, Smętna 12, 31-343 Kraków, Poland

**Keywords:** Levomepromazine, Clozapine, Cytochrome P450, Induction, Human hepatocytes

## Abstract

**Background:**

Cytochrome P450 (CYP) enzymes are involved in the metabolism of many important endogenous substrates (steroids, melatonin), drugs and toxic xenobiotics. Their induction accelerates drug metabolism and elimination. The present study aimed at examining the inducing abilities of two antipsychotic drugs levomepromazine and clozapine for the main CYPs.

**Methods:**

The experiments were performed using cryopreserved human hepatocytes. The hepatotoxicity of levomepromazine and clozapine was assessed after exposure to the neuroleptics (LDH test). CYP activities were measured in the incubation medium using the CYP-specific reactions: caffeine 3-*N*-demethylation (CYP1A1/2), diclofenac 4′-hydroxylation (CYP2C9), perazine *N*-demethylation (CYP2C19) and testosterone 6β-hydroxylation (CYP3A4). In parallel, *CYP* mRNA levels were measured in neuroleptic-treated hepatocytes.

**Results:**

The results indicate that levomepromazine and clozapine induce the expression of main CYP enzyme CYP3A4 in human hepatocytes. Levomepromazine and clozapine at concentrations of 2.5 and 10 µM, respectively, caused a significant increase in the mRNA level and activity of CYP3A4. Both neuroleptics did not produce any changes in CYP1A1/2, CYP2C9 and CYP2C19.

**Conclusion:**

Levomepromazine and clozapine induce CYP3A4 in human hepatocytes in vitro. Further in vivo studies are advisable to confirm the CYP3A4 induction by levomepromazine and clozapine in the liver, and to assess the effect of these drugs on their own metabolism and on the biotransformation of other co-administered drugs which are the CYP3A4 substrates.

## Introduction

Cytochrome P450 (CYP) is one of the largest drug-metabolizing enzyme systems whose expression has significant substrate and tissue specificity. The CYP enzymes are involved in the biotransformation of most drugs and other xenobiotics, and help to eliminate them out of the body [1]. Moreover, CYPs regulate many important life processes, such as the metabolism of melatonin, steroids (e.g., biosynthesis of estrogen), arachidonic acid, conversion of cholesterol into bile acids and biotransformation of bile acids.

Levomepromazine is a typical phenothiazine antipsychotic with sedative/hypnotic, anxiolytic, antiemetic, analgesic and antipsychotic activities. It is used for treating schizophrenia, paranoia, mania, toxic psychosis and mental organic syndromes associated with delirium [2]. Clozapine was the first second-generation antipsychotic released for clinical use. It is mainly used for schizophrenia patients who do not improve following treatment with other antipsychotic medications [3].

The knowledge of the ability of levomepromazine and clozapine to induce CYP enzymes would be of pharmacological and clinical importance, since these drugs are administered to patients for months or years, and very often in combination with other drugs, which are substrates of the CYP enzymes. Although there are some experimental data suggesting a possibility of induction of CYP isoforms by typical and atypical neuroleptics [4], the ability of levomepromazine and clozapine to induce human CYPs has not been studied so far. Levomepromazine is chiefly metabolized by CYP3A4 via 5-sulfoxidation and *N*-demethylation [5], while clozapine is mainly biotransformed by CYP1A2 via *N*-demethylation and by CYP3A4 via *N*-oxidation [6].

The aim of this work was to ascertain whether levomepromazine and clozapine, the neuroleptics with different chemical structures and pharmacological profiles, may induce CYP1A1/2, CYP2C9, CYP2C19 and CYP3A4 enzymes in human liver.

## Materials and methods

### Drugs and chemicals

All media for culture of human hepatocytes, PBS Buffer, LDH Cytotoxicity Detection Kit were purchased from ThermoFisher Scientific (Waltham, MA, USA). Levomepromazine was obtained from Egyt (Budapest, Hungary), clozapine from Anpharm (Warszawa, Poland). Trypan Blue, dimethyl sulfoxide (DMSO), rifampicin, β-naphthoflavone, TRI Reagent, caffeine, paraxanthine, diclofenac, 4-hydroxydiclofenac, and *N*-desmethylperazine were purchased from Sigma-Aldrich (St.Louis, MO, USA). Perazine was obtained from Labor (Wrocław, Poland). Testosterone and its metabolites were provided by Steraloids (Newport, KY, USA). All of the organic solvents with HPLC purity were supplied by Merck (Darmstadt, Germany).

### Cell culture

Experiments were performed in vitro using inducible-qualified human cryopreserved hepatocytes from three different lots. Cryopreserved human hepatocytes constitute a universally accepted experimental in vitro system for the evaluation of drug metabolic properties in human liver. Human hepatocyte donors (HU1836, HU1663 and HU1832, from ThermoFisher Scientific, Waltham, MA, USA) were thawed in hepatocyte recovery medium, viability was determined using Trypan Blue, according to the manufacturer’s protocols. The viability of the cell suspensions was > 90%. Hepatocytes were seeded in collagen type-I-coated 48-well plates from Corning (NY, USA), at a density of 1.35 × 10^5^ viable cells in 0.25 ml of plating medium for each well in Williams Medium E. The plates were incubated at 37 °C in 5% CO_2_ with saturating humidity to allow cells to adhere to the plate. The neuroleptics and positive inducers were added to the culture medium in 0.1% DMSO at a concentration of 0.25, 0.75, 2.5 µM for levomepromazine, 1, 2.5, 10 µM for clozapine, 25 µM for rifampicin (CYP2C9, CYP2C19 and CYP3A4 inducer), and 20 µM for β-naphthoflavone (CYP1A1/2 inducer). 0.1% DMSO was used as a vehicle control. The above procedure simulates the conditions of long-term treatment in the clinic. For each lot of hepatocytes, five separate wells per treatment conditions were used. The hepatocytes were incubated for 72 h with a daily replacement of the culture medium containing one neuroleptic or one inducer (a positive control).

### LDH (lactic acid dehydrogenase) assay

Thawed human hepatocytes were seeded in collagen type-I-coated 96-well plates in 100 µl of incubation medium at conditions described above. After 24 h, the hepatocytes were treated with 0.01, 0.1, 0.5, 1, 2.5, 5, 10, 25, 50, 100 and 200 µM of levomepromazine or clozapine in 0.1% DMSO (0.1% DMSO served as a control). At the end of the treatment, 50 µl of medium solution from all wells was transferred to a 96-well plate and the LDH reaction was performed using Pierce LDH Cytotoxicity Assay Kit following the manufacturer’s instructions.

### CYP activity assay

After 72-h incubation of hepatocytes with the tested neuroleptics or inducers, the culture media were changed to a medium without the neuroleptics or inducers, but containing the CYP enzyme-specific substrates to assess specific CYP activities. After the incubation for 60 min, 100 µl of the culture medium from each well was transferred into Eppendorf tubes containing 10 µl of acetonitrile. CYP enzyme activities were determined in the culture medium using the following CYP-specific reactions: caffeine 3-*N*-demethylation (CYP1A1/2), diclofenac 4′-hydroxylation (CYP2C9), perazine *N*-demethylation (CYP2C19) and testosterone 6β-hydroxylation (CYP3A4). The concentrations of CYP-specific substrates and their metabolites formed in the culture medium were measured by the HPLC method with UV detection, as described previously [7].

### Determination of mRNA level

After incubation with the tested compounds, the cells were washed with ice-cold PBS buffer, scraped and collected by centrifugation (5 min, 300×*g*). Total cellular RNA was prepared using TRI Reagent method, according to the manufacturer’s instructions. cDNA was synthesized from total RNA using a Transcriptor High Fidelity cDNA Synthesis Kit (Roche) according to the manufacturer’s instructions. The cDNA was used as a template for qPCR using TaqMan Gene Expression Assays: CYP1A1 (Hs01054797_g1), CYP1A2 (Hs00167927_m1), CYP2C9 (Hs02383631_s1), CYP2C19 (Hs0042638_m1), CYP3A4 (Hs00604506_m1), β-actin (Hs99999903_m1). The expression levels of *CYP1A1, CYP1A2, CYP2C9, CYP2C19* and *CYP3A4* were normalized to *β-actin* and calculated using 2-delta Ct method as described previously [8].

### Statistical data analysis

The obtained values are the mean of five replicated experiments ± SEM. Statistical analysis of activity, LDH and mRNA levels was performed using one-way analysis of variance (ANOVA) followed by Dunnett’s post hoc test (GraphPad Prism 7.0). The results were regarded as statistically significant when *p* < 0.05.

## Results

### Cytotoxic effects of levomepromazine and clozapine

**Fig. 1:**
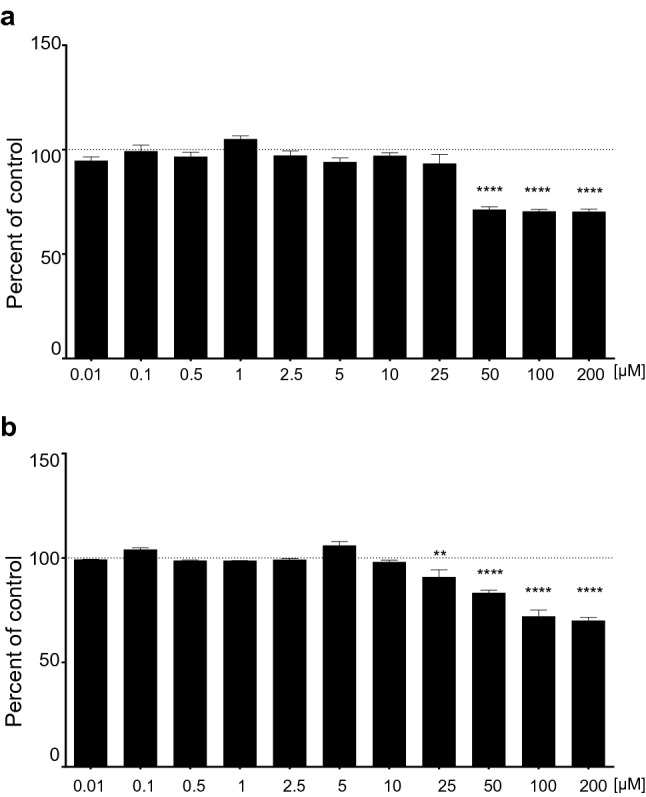
Viability of cryopreserved human hepatocyte after treatment with a series of concentrations (0.01–200 µM) of levomepromazine **a** or of clozapine **b**. The results are shown as the percent of control (mean ± SEM, *n* = 3). One-way ANOVA, Dunnett’s test: **p < 0.01 and ****p < 0.0001 versus respective control

The hepatotoxicity of levomepromazine and clozapine on cryopreserved human hepatocytes was routinely assessed by estimating cellular damage by measuring lactate dehydrogenase (LDH) release into the culture medium. Cytotoxicity of levomepromazine and clozapine was expressed as the percent of control (0.1% DMSO-treated hepatocytes) (Fig. 1a, b). The obtained results showed that 0.1% DMSO did not affect hepatocyte culture (data not shown), while the concentrations of levomepromazine above 50 µM and of clozapine above 25 µM were toxic for hepatocytes. Lower concentrations of both neuroleptics (0.01–25 µM for levomepromazine and 0.01–10 µM of clozapine) could be managed by hepatocytes (Fig. 1a, b).

### CYP enzyme activities in cryopreserved human hepatocytes

**Fig. 2:**
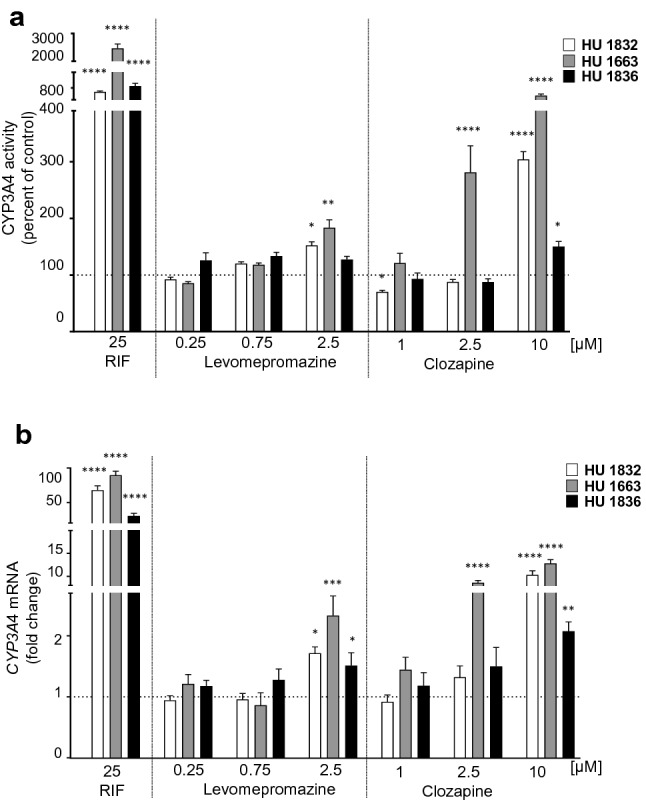
The effect of rifampicine, levomepromazine and clozapine on the CYP3A4 activity and mRNA level in cryopreserved human hepatocytes from three different lots. **a** CYP3A4 activity level was measured as the rate of testosterone 6β-hydroxylation and presented as the percent of control. The control values for CYP3A4 activity (pmol/mg protein/min) were: 0.13 ± 0.01 for HU1832, 0.04 ± 0.004 for HU1663 and 0.07 ± 0.001 for HU1836. **b** The *CYP3A4* mRNA level was presented as fold change upon drug treatment (mean ± SEM, *n* = 5). One-way ANOVA (see Table 1), Dunnett’s test: *p < 0.5; **p < 0.01; ***p < 0.001; ****p < 0.0001 versus respective control

The effects of the tested drugs (levomepromazine and clozapine) and inducers on CYP enzyme activities were evaluated in three human hepatocyte donors. 0.1% DMSO did not affect CYP enzyme activities (data not shown). Rifampicin potently raised the CYP3A4 activity in all three donors (Fig. 2a, Table 1). In parallel, clozapine at the concentration of 10 µM significantly increased the CYP3A4 activity up to 304% for HU1832, 605% for HU1663 and 150% for HU1836. Levomepromazine at a concentration of 2.5 µM enhanced the CYP3A4 activity up to 152% for HU1832, 183% for HU1663 and 127% for HU1836 (Fig. 2a), but lower concentrations of both tested neuroleptics (levomepromazine: 0.25 and 0.75 µM, clozapine: 1 and 2.5 µM) did not produce any significant changes in the CYP3A4 activity, only 2.5 µM clozapine concentration caused the HU1663 hepatocyte donor to increase it to 281% (Fig. 2a, Table 1).

**Table 1:** Summary of the one-way analysis of variance (one-way ANOVA) for the effects of the antipsychotic drugs on the hepatocytes’ viability (Fig. 1a, b), CYP3A4 activity (Fig. 2a) and mRNA levels (Fig. 2b)

Group	*F*	*df*	*p* value
Figure 1a Levomepromazine 0.01–200 μM	35.36	11.24	** < 0.0001**
Figure 1b Clozapine 0.01–200 μM	56.18	11.24	** < 0.0001**
Figure 2a			
RIF 25 μM	86.71	3.16	** < 0.0001**
Levomepromazine 0.25 μM	4.401	3.16	**0.0263**
Levomepromazine 0.75 μM	5.362	3.16	**0.0107**
Levomepromazine 2.5 μM	8.265	3.16	**0.0025**
Clozapine 1 μM	7.484	3.16	**0.0037**
Clozapine 2.5 μM	57.29	3.16	** < 0.0001**
Clozapine 10 μM	185.7	3.16	** < 0.0001**
Figure 2b			
RIF 25 μM	72.98	3.16	** < 0.0001**
Levomepromazine 0.25 μM	1.502	3.16	0.2522
Levomepromazine 0.75 μM	1.529	3.16	0.2454
Levomepromazine 2.5 μM	9.103	3.16	**0.0009**
Clozapine 1 μM	1.629	3.16	0.2223
Clozapine 2.5 μM	9.525	3.16	**0.0008**
Clozapine 10 μM	107.6	3.16	** < 0.0001**

Bold indicates effects that are significant (*p* < 0.05)

Moreover, β-naphthoflavone potently enhanced the activity of CYP1A1/2 and rifampicin that of CYP2C9 and CYP2C19, while levomepromazine and clozapine did not significantly affect the activity of those enzymes at any concentration tested in any of the evaluated human hepatocyte donors (data not shown).

### Effect of levomepromazine and clozapine on CYP mRNA levels in human hepatocytes

In parallel with the activity changes, rifampicin potently raised the *CYP3A4* mRNA level in all the three donors (Fig. 2b, Table 1). Levomepromazine at the concentration of 2.5 µM caused a 1.7-, 2.3- and 1.5-fold induction of CYP3A4 mRNA in hepatocyte donors HU1832, HU1663 and HU1836, respectively (Fig. 2b, Table 1). Clozapine at the concentration of 10 µM turned out to be a stronger inducer than levomepromazine and caused a 5.4-, 7.9- and 2.1-fold induction of *CYP3A4* mRNA in hepatocyte donors HU1832, HU1663 and HU1836, respectively (Fig. 2b, Table 1). Lower concentrations of both tested neuroleptics (levomepromazine: 0.25 and 0.75 µM, clozapine: 1 and 2.5 µM) did not produce any significant changes in the *CYP3A4* mRNA level, but 2.5 µM clozapine concentration resulted in 3.6-fold induction of *CYP3A4* mRNA in the HU1663 hepatocyte donor (Fig. 2b, Table 1).

Besides, β-naphthoflavone potently enhanced the level of *CYP1A1/2* mRNA and rifampicin that of *CYP2C9* and *CYP2C19*, while levomepromazine and clozapine did not affect the mRNA levels of those genes in any of the donors (data not shown).

The results of the statistical analysis are summarized in Table 1.

## Discussion

The present study indicates that levomepromazine and clozapine induce the expression of CYP3A4 enzyme in human hepatocytes. Levomepromazine at a concentration of 2.5 µM and clozapine at a concentration of 10 µM caused significant increase in the CYP3A4 mRNA and activity level. Both neuroleptics at the tested concentrations did not produce any changes in the expression and activity of CYP1A1/2, CYP2C9 and CYP2C19 enzymes. It suggests that the neuroleptics tested do not act as direct ligands of the nuclear receptors PXR or CAR, which are engaged in the transcriptional regulation of both *CYP3A4* and *CYP2C* genes [9].

It is worth noting that levomepromazine and clozapine at the concentrations tested for *CYP* induction did not cause any cytotoxic effect on the cryopreserved human hepatocytes. But the concentrations of levomepromazine above 50 µM and of clozapine above 25 µM were toxic for hepatocytes. Similar level of clozapine hepatotoxicity was observed by Magliaroa and Saldanha [10] and Lu and co-workers [11]. On the other hand, treatment of hepatocytes with the specific inducers β-naphthoflavone or rifampicin [12, 13] significantly increased the mRNA levels of all the tested genes (*CYP1A1/2* or *CYP2C9, CYP2C19* and *CYP3A4,* respectively) in all the tested hepatocyte donors, suggesting that the hepatocytes from different donors used in the present study retained the regulation mechanisms of *CYP* expression.

As mentioned above, levomepromazine and clozapine increased the expression and activity of CYP3A4 at the highest concentrations tested (2.5 µM and 10 µM, respectively). These concentrations are higher than the therapeutic ones in the blood plasma (up to 0.5 µM and 1.8 µM, respectively) [14, 15]. However, during a long-term pharmacotherapy, their concentrations in the liver may be several times higher than in plasma owing to their physicochemical properties and consequent cell and tissue distribution pattern. Basic lipophilic drugs accumulate in cellular membranes and are taken up by acidic compartments (lysosomes), which are abundant in the liver [16]. It is, therefore, possible that CYP3A4 induction observed in vitro will also occur in patients showing higher plasma therapeutic concentrations*,* and will lead to pharmacokinetic interactions.

Our previous experiment performed in vitro using the human cDNA-expressed CYP3A4 enzyme showed that levomepromazine mildly inhibited the activity of CYP3A4 (testosterone 6β-hydroxylation) via a mixed mechanism (*K*_i_ = 34 μM) [7]. Thus, levomepromazine may exert its action on the CYP3A4 enzyme via two different mechanisms: direct inhibition of protein activity, as shown for human cDNA-expressed CYP3A4 [7] and classical enzyme induction, as demonstrated in the present work using human hepatocytes at a neuroleptic concentration of 2.5 µM. The latter effect (enzyme induction) may be expected to prevail in vivo, since it occurs at a lower neuroleptic concentration, as shown the above-presented in vitro studies. Thus, both kinds of experimental approaches are advisable to obtain the whole picture of drug effect on enzyme, i.e. the application of human cDNA-expressed CYP enzymes or liver microsomal fractions to observe a direct effect on enzyme, and the utilization of human hepatocytes (fresh or cryopreserved hepatocytes or cell lines) to evoke a possible effect on *CYP* gene expression, such as enzyme induction.

A few clinical reports suggest that levomepromazine can induce cytochrome P450. A significant decrease in the plasma levels of clozapine or quetiapine was indeed observed in psychiatric patients simultaneously treated with levomepromazine [17, 18]. Since quetiapine is metabolized chiefly by CYP3A4 and this enzyme is partly involved in the metabolism of clozapine [19], it seems feasible that the changes observed in the plasma levels of these drugs may result from CYP3A4 induction by levomepromazine. On the other hand, CYP3A induction by clozapine was observed in rat liver [20]. Since these neuroleptics may be administered for months or even years, also to patients treated simultaneously with other clinically important medications that are substrates of CYP3A4 (e.g., antidepressants, carbamazepine, cyclosporin A, calcium channel antagonists, macrolide antibiotics) [21, 22], the tested drugs may enhance the metabolism of the co-administered drugs, leading to the diminution of their pharmacological effect. In addition, hepatic CYP3A4 induction by levomepromazine or clozapine may alter the metabolism of endogenous substrates (e.g., steroids), contributing to side-effects. The direct evidence of the potential of levomepromazine and clozapine to induce CYP3A4 isoenzyme, obtained in the present study, constitutes the basis for further clinical in vivo studies. The in vivo application of specific CYP3A4 marker substrates before and after neuroleptic treatment would provide an answer on the final effect of those drugs on the enzyme activity and allow assessing the probability of drug–drug interactions.

## Conclusion

The obtained results show that levomepromazine and clozapine at the concentrations of 2.5 µM and 10 µM, respectively, increase the expression and activity of the main drug-metabolizing enzyme CYP3A4 in human hepatocytes. Since these neuroleptics can reach such concentrations in the liver during a long-term therapy of psychiatric patients, they may increase the CYP3A4 activity in clinical conditions. Further in vivo studies are advisable to confirm the CYP3A4 induction by levomepromazine and clozapine in the liver, and to assess the effect of these drugs on their own metabolism and on the biotransformation of other co-administered drugs which are the CYP3A4 substrates.
